# Improving transient expression in *N. benthamiana* by suppression of the *Nb-SABP2* and *Nb-COI1* plant defence response related genes

**DOI:** 10.3389/fpls.2024.1453930

**Published:** 2024-09-09

**Authors:** Lilya Kopertekh

**Affiliations:** Institute for Biosafety in Plant Biotechnology, Julius Kühn-Institut (JKI) - Federal Research Centre for Cultivated Plants, Quedlinburg, Germany

**Keywords:** *Nicotiana benthamiana*, *Nb-COI1*, *Nb-SABP2*, plant defense response, recombinant proteins, transient expression

## Abstract

Currently transient expression is one of the preferred plant-based technologies for recombinant protein manufacturing, particularly in respect to pharmaceutically relevant products. Modern hybrid transient expression systems combine the features of *Agrobacterium tumefaciens* and viral vectors. However, host plant reaction to Agrobacterium-mediated delivery of gene of interest can negatively affect foreign protein accumulation. In this study, we investigated whether the modulation of plant immune response through knockdown of the *Nb-SABP2* and *Nb-COI1 N. benthamiana* genes could improve recombinant protein yield. In plants, the SABP2 and COI1 proteins are involved in the salicylic acid and jasmonic acid metabolism, respectively. We exemplified the utility of this approach with the green fluorescence (GFP) and β nerve growth factor (βNGF) proteins: compared to the tobacco mosaic virus (TMV)-based vector the *Nb-SABP2* and *Nb-COI1*-suppressed plants provided an increased recombinant protein accumulation. We also show that this strategy is extendable to the expression systems utilizing potato virus X (PVX) as the vector backbone: the enhanced amounts of βNGF were detected in the *Nb-SABP2* and *Nb-COI1*-depleted leaves co-infiltrated with the PVX-*βNGF*. These findings suggest that modulating host plant reaction to agrodelivery of expression vectors could be useful for improving transient foreign protein production in *N. benthamiana*.

## Introduction

Transient expression in *N. benthamiana* is an advanced technology, which has turned plants into commercial platform for recombinant protein production (Lomonossoff and D’Aoust, 2016; Schillberg et al., 2019; Schillberg and Finnern, 2021). This method uses the ability of *Agrobacterium tumefaciens* to transfer T-DNA carrying the expression vector with the gene of interest into plant cells (Kapila et al., 1997; Fischer et al., 1999). The major advances in this technique have centered on the development of hybrid expression systems combining features of *A. tumefaciens* and viral vectors (Gleba et al., 2005; Peyret and Lomonossoff, 2015). With a few exceptions the modern viral expression vectors rely on sequences of RNA (tobacco mosaic virus (TMV), potato virus X (PVX), cowpea mosaic virus (CPMV)) and DNA (bean yellow dwarf virus (BeYDV)) viruses (Gleba et al., 2007; Peyret and Lomonossoff, 2015). Among different species that have been tested for transient expression *Nicotiana benthamiana* is the preferred host due to a short life cycle, fast growth rate, suitable for massive infiltration leaf anatomy and a partly defective RNA silencing system (Goodin et al., 2008; Bally et al., 2018). Current research in the field of *N. benthamiana*-based transient expression is focused on the improvement of vector delivery systems and fine-tuning of plant expression host. A number of strategies have been developed to design plant cell conditions supporting foreign protein production. The first one is based on co-expression of cell cycle regulatory genes. In particular, several research groups reported about positive impact of virus- and plant-derived cell-cycle regulators on recombinant protein accumulation in *N. benthamiana* (Norkunas et al., 2018; Kopertekh and Reichardt, 2021, 2022). Another experimental approach of plant cell engineering has been described in publication of Wang et al. (2023). The authors showed that the application of a semi-dominant negative gain-of-function mutant form of plasmodesmata located protein 5 (PDLP) from *Arabidopsis thaliana* facilitated TMV-based vector movement and enhanced foreign protein production. One of the recent reports demonstrated that the depletion of the NbCORE receptor perceiving the cold shock protein of *A. tumefaciens* improves agroinfiltration productivity in *N. benthamiana* (Dodds et al., 2023). Finally, the attenuation of plant defense response against microbial components of transient vector delivery system has been also addressed to design a supportive cell environment for increased foreign protein accumulation. There are several examples in the literature showing that downregulation of endogenous plant genes involved in post-transcriptional gene silencing (PTGS) process can elevate recombinant protein accumulation in *N. benthamiana* host. Particularly, this approach has been shown to be feasible for the *DCL2*, *DCL4* (Matsuo and Matsumura, 2017; Matsuo, 2022), *RDR6* (Matsuo and Atsumi, 2019) and *Arg2* (Ludman et al., 2017) genes encoding Dicer-like 2, Dicer-like 4, RNA-dependent RNA polymerase 6 and Argonaut 2 proteins, respectively.

One of the essential components of the plant pathogen response is the regulatory network of phytohormones. Salicylic acid (SA), jasmonic acid (JA) and ethylene (ET) are major plant hormones that orchestrate defense reaction (Li et al., 2019; Ngou et al., 2022). The phytohormones involved in plant response to biotic stress have been shown to play an essential role in both Agrobacterium- and virus-plant interactions. In respect to plant-Agrobacterium pathosystem it was demonstrated that the defective in SA accumulation *Arabidopsis thaliana* and *N. bethamiana* plants were more susceptible to infection, whereas mutants overproducing SA were relatively resistant (Yuan et al., 2007; Anand et al., 2008). It was suggested that SA shuts down the expression of the Vir regulon by the attenuation of the VirA protein kinase activity (Yuan et al., 2007). The role of JA and SA in plant-virus interaction has also been reported. For example, the exogenous application of SA and JA improved plant resistance to RNA viruses (Lee et al., 2011; Shang et al., 2011). Inversely, suppression of the SA and JA biosynthetic and signaling genes accelerated development of symptoms and accumulation of virus titters (Liu et al., 2004; Pacheco et al., 2012; García-Marcos et al., 2013; Zhu et al., 2014). Considering the negative impact of plant defense response on microbial component of transient vector delivery system, we supposed that the attenuation of defense reaction might have positive effect on agroinfiltration efficiency and subsequent recombinant protein accumulation. To proof our hypothesis we modulated pathogen defense in *N. benthamiana* by silencing two endogenous genes, *Nb-SABP2* and *Nb-COI1*, which are involved in SA and JA metabolism, respectively. The *Nb-SABP2* gene encodes a SA-binding protein 2 termed SABP2, which is essential for establishing systemic acquired resistance (SAR) (Kumar and Klessig, 2003; Forouhar et al., 2005). The SABP2 is involved in the conversion of biologically inactive methyl salicylate (MeSA) into active SA leading to activation of the SA-dependent defense response (Park et al., 2007). The coronatine insensitive 1 (*COI1*) gene encodes an F-box COI1 protein that determines the substrate specificity of the E3 ubiquitin ligase SCF^COI1^ complex. This complex targets repressors of the JA-induced genes for degradation inducing their expression (Xie et al., 1998; Xu et al., 2002; Chini et al., 2009).

We report here that transient downregulation of the *Nb-SABP2* and *Nb-COI1* genes facilitated accumulation of viral vector and target protein transcripts, and resulted in enhanced recombinant protein production in *N. benthamiana*. These findings indicate that the attenuation of plant host response against biotic stress could have value in improving transient expression technology.

## Materials and methods

### Plasmid constructs

The pLH-*35S-COI1-INT-COI1* and pLH-*35S-SABP2-INT-SABP2* constructs were designed in several steps. The 186 bp region of the *Nb-COI1* gene was amplified from *N. benthamiana* cDNA using two primer combinations, NcoI-COI1-forw/COI1-BamHI-rev and SpeI-COI1forw/COI1-XhoI-rev. The 230 bp fragment of the *Nb-SABP2* gene was amplified from the same cDNA sample as for the *Nb-COI1* gene by two primer pairs: the NcoI-SABP2-forw/SABP2-HindIII-rev and the XbaI-SABP2-forw/SABP2-SalI-rev. The second intron (IV2) of the potato *ST-LS1* gene was amplified from the p35S-creINT plasmid (Mlynárová and Nap, 2003) by PCR using the BglII-ST-LS1-INT-forw/ST-LS1-INT-SalI-rev and HindIII-ST-LS1-INT-forw/STLS1-INT-SalI-rev primer combinations. The amplified fragments were cloned in pGEM-T Easy (Promega, Walldorf, Germany) vector and sequenced. The primers can be found in **Supplementary Table S1**. Next, the sense and antisense gene fragments together with the separating hairpin loop sequence were introduced between the 35S promoter and terminator in the pCK-*GFP* plasmid (Reichel et al., 1996). The pCK-*35S-COI1-INT-COI1* construct was generated by the ligation of the *Nco*I-*Xba*I digested pCK-*GFP* with three restriction fragments, NcoI-COI1-BamHI, BglII-ST-LS1-INT-SalI and SpeI-COI1-XhoI, derived from the pGEM-*NcoI-COI1-BamHI*, pGEM-*BglII-ST-LS1-INT-SalI* and pGEM-*SpeI-COI1-XhoI* plasmids, respectively. The restriction enzymes used for cloning are defined at 5’ and 3’ ends of the restriction fragments. Finally, the Nb-COI1 RNAi cassette from the pCK-*35S-COI1-INT-COI1* plasmid was cloned into *Pst*I restriction site of the pLH7000 binary vector (Töpfer et al., 1993) resulting in the pLH-*35S-COI1-INT-COI1* (*Nb-COI1hpc*).

Ligation of the *Nco*I/*Xba*I digested pCK-*GFP* plasmid with three restriction fragments, NcoI-SABP2-HindIII, HindIlI-ST-LS1-INT-SalI and XbaI-SABP2-SalI, yielded the pCK-*35S-SABP2-INT-SABP2* plasmid. For the *Nb-SABP2* gene, the NcoI-SABP2-HindIII, HindIlI-ST-LS1-INT-SalI and XbaI-SABP2-SalI restriction fragments were obtained from the pGEM-*NcoI-SABP2-HindIII*, pGEM-*HindIlI-ST-LS1-INT-SalI* and pGEM-*XbaI-SABP2-SalI* plasmids. Next, the silencing expression cassette from the pCK-*35S-SABP2-INT-SABP2* intermediate construct was released by *Pst*I and ligated to *Nsi*I digested pLH7000 resulting in the pLH-*35S-SABP2-INT-SABP2* (*Nb-SABP2-hpc*) final plasmid.

To design the pLH-*TMV-GFP* expression construct the *Hind*III-*Kpn*I digested pLH-Δbar-*Pac*I (Kopertekh et al., 2004) plasmid was ligated with three restriction fragments, namely, *Kpn*I*-Nco*I fragment of pICH17344, *Nco*I-*Spe*I fragment of pICH17344, and *Spe*I-*Hind*III fragment of pICH17344. The pLH-*TMV-GFP* was subsequently used to prepare the pLH-*TMV-βNGF*. At first, the *βNGF* gene containing *Xho*I and *Swa*I restriction sites at 5’ and 3’ ends, respectively, was synthesized by BioCat (Heidelberg, Germany). Then, *Xho*I-*β-NGF-SwaI* sequence digested with *Xho*I and *Swa*I enzymes was introduced into identical cloning sites of the pLH-*TMV-GFP* construct.

The pLH-*PVX-βNGF* expression vector was constructed as follows. At first, the *βNGF* gene containing *Nhe*I restriction site at the 5’ of the start codon and *Sal*I restriction site at the 3’ end of the stop codon was synthesized by BioCat (Heidelberg, Germany). Following *Nhe*I-*Sal*I restriction the *βNGF* sequence was ligated into equally digested p*PVX201* (Chapman et al., 1992) producing the p*PVX-βNGF*. To transfer the *PVX-βNGF* sequence into the pLH*-Δbar-PacI* plasmid the p*PVX-βNGF* was linearized with *Sph*I and, following removal of the single stranded termini with T4 polymerase digested with *Ehe*I. The *Sph*I-*Ehe*I DNA fragment containing the *PVX-βNGF* sequence was ligated to pLH*-Δbar-PacI* plasmid restricted with *Stu*I. The final plasmid was designated as pLH-*PVX-βNGF.*

The pLH7000, pLH-35S-*COI1-INT-COI1*, pLH-*35S-SABP2-INT-SABP2*, pLH-*TMV-GFP*, pLH-*TMV-βNGF* and pLH-*PVX-βNGF* expression vectors were introduced into *A. tumefaciens* (recently renamed to *Rhizobium radiobacter*) strain C58C1 by the freeze-thaw method (Holsters et al., 1978).

### Agroinfiltration of *N. benthamiana*

*A. tumefaciens* cultures (strain C58C1) containing the expression vectors were grown overnight in LB medium supplemented with appropriate antibiotics (100 mg/L spectinomycin, 300 mg/L streptomycin, 50 mg/L rifampicin, 100 mg/L carbenicillin), pelleted by centrifugation and resuspended in agroinfiltration buffer (10 mM MES, 10 mM MgCl_2_, 150 µM acetosyringone, pH 5.6). The final optical density of *A. tumefaciens* cultures carrying the pLH-*TMV-GFP*, pLH-*TMV-βNGF* and pLH-*PVX-βNGF* vectors was adjusted to OD600 of 0.1, whereas the *A. tumefaciens* cultures carrying the pLH7000, pLH-*35S*-*COI1-INT-COI1* and pLH-*35S-SABP2-INT-SABP2* plasmids were brought to a final OD600 of 0.3. Agrobacterium suspensions were infiltrated into expanded leaves of 5-6 week-old *N. benthamiana* plants using a syringe without a needle. *N. benthamiana* plants were grown in the greenhouse at 24/22 ^0^C day/night temperature with a 16 h light and 8 h dark photoperiod.

### Gene expression analysis

Expression of the *Nb-SABP2*, *Nb-COI1*, TMV, *GFP* and *βNGF* was estimated by qPCR analysis. To this end, RNA from the agroinfiltrated and non-agroinfiltrated *N. benthamiana* plants was isolated using the BioSELL RNA Mini Kit (Bio&SELL, Feucht, Germany). One μg of RNA was used to synthesize cDNA by the random hexamer primer and Maxima Reverse Transcriptase according to the manufacturer’s protocol (Thermo Scientific, Waltham, USA). The qPCR has been carried out with the Nb-SABP2-forw/Nb-SABP2-rev, Nb-COI-forw/NbCOI-rev, gfp-forw/gfp-rev, βNGF-forw/βNGF-rev and TMV-CP-forw/TMV-CP-rev primers specific to the *Nb-SABP2* (JX317629.1), *Nb-COI1* (AY547493.1), *GFP* (KX458.181.2), *βNGF* (KF057035.1) and TMV coat protein (M34077.1) genes, respectively. The qPCR reactions were accomplished in Mastercycler^®^ EP realplex (Eppendorf, Hamburg, Germany) utilizing Maxima SYBR Green qPCR Master Mix (Thermo Scientific, Waltham, USA). Relative quantifications were performed based on the ΔCT method using cyclophilin (*cyp*) (AY368274.1) gene as an internal standard. Primer sequences for target and reference genes are listed in **Supplementary Table S1**.

In total samples from 6-8 plants were subjected to RNA analysis. Each sample was pooled from three middle leaves of one plant. The number of samples is designated in figure legends as n. Three technical replicates were performed for each probe. The Mann-Whitney U test was applied to test if there were differences between wild type and silenced plants.

### GFP imaging

For visual detection of GFP fluorescence *N. benthamiana* leaves were illuminated with a handheld UV lamp and photographed with the Canon digital camera EOS 300D.

### Protein quantification

The GFP and βNGF proteins were quantified by specific ELISA. Plant samples were collected from the middle agroinfiltrated leaves at 3, 5 and 7 dpi. The description of the GFP quantification can be found in Kopertekh and Reichardt (2021). The amounts of the βNGF protein in *N. benthamiana* leaves were determined as follows. Leaf material (300 mg) was harvested, homogenized in two volumes (w/v) of TBS buffer (50 mM Tris, 150 mM NaCI, 0.05% Tween-20, pH 7.4) and clarified by centrifugation for 10 min at 4 °C. ELISA plates were coated overnight with capture antibody diluted in carbonate buffer (0.1 M NaHCO_3_, 0.1 M Na_2_CO_3_, pH 9.5). After incubation, the plates were washed three times with TBS buffer and blocked with blocking solution (TBS buffer supplemented with 1% BSA) for 1 h at room temperature. Following washing step as described above the plates were coated with the fresh prepared plant extracts and incubated overnight at 4 °C. Subsequently, the washing with TBS was repeated and a biotin anti-human NGF antibody in TBS buffer containing 1% BSA was added and incubated for 1 h at room temperature. After washing step performed as described, an avidine alkaline phosphatase was added and incubated for 30 minutes at room temperature. Finally, the plates were developed for 30 min with p-nitrophenyl phosphate as substrate and optical density was measured at 405 nm in a SUNRISE™ microplate reader (Tecan, Männedorf, Switzerland). All plates contained control βNGF protein diluted in TBS buffer for a standard curve.

Recombinant protein accumulation was analyzed in 6-8 biological and three technical replicates. The biological replicate represents a pooled sample from three middle leaves of one plant. The Mann-Whitney U test was performed to test if there were differences in recombinant protein accumulation between agroinfiltrated wild type and silenced plants.

## Results

### Expression of the *Nb-SABP2* and *Nb-COI1* genes in agroinfiltrated *N. benthamiana* leaves

**Figure 1:**
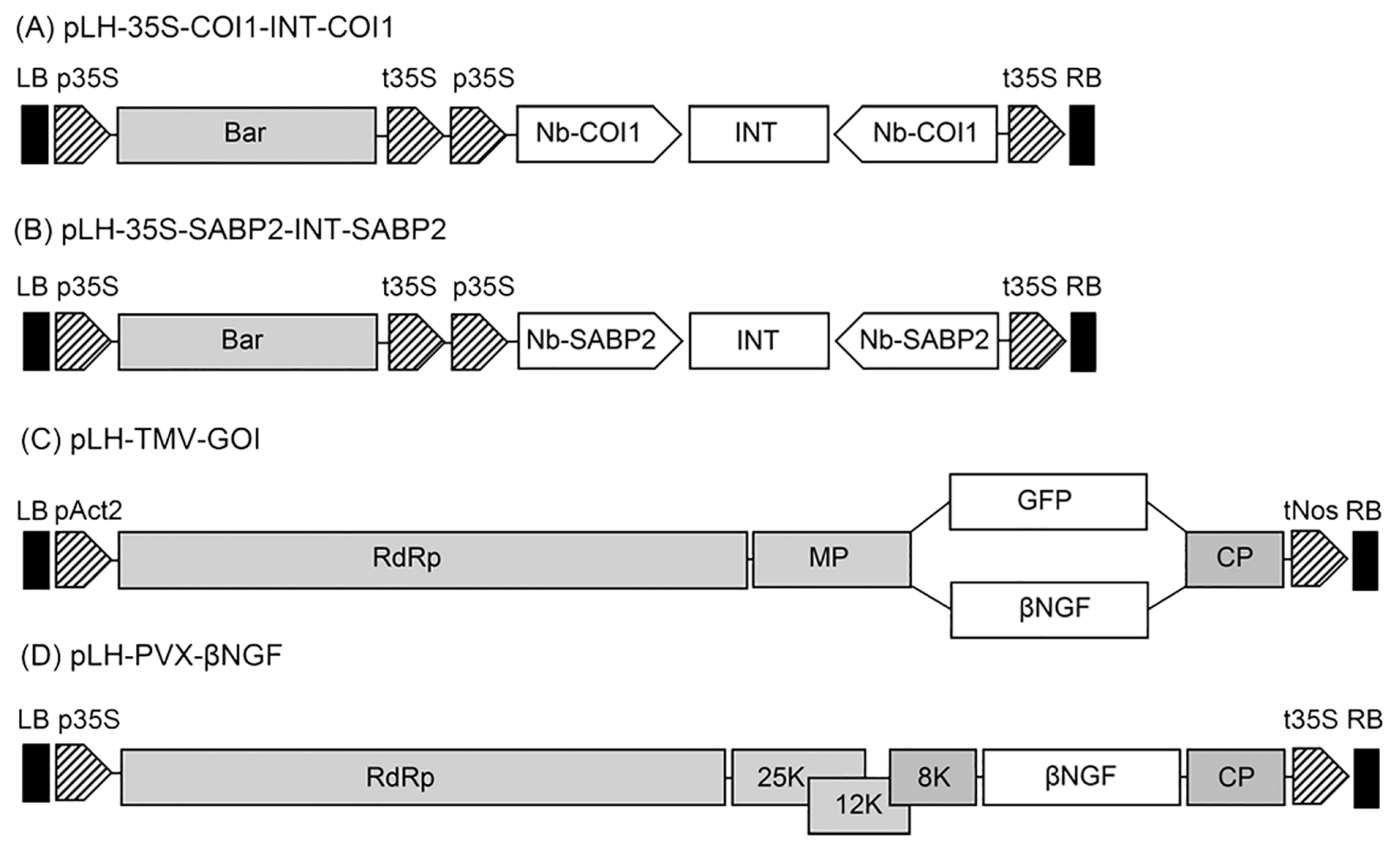
Schematic representation of expression constructs used in this study. **(A)** The pLH-*35S-COI1-INT-COI1* RNAi construct. The pLH-*35S-COI1-INT-COI1* plasmid contains the *bar* and *Nb-COI1* RNAi expression cassettes. The components of the pLH-*35S-COI1-INT-COI1* are designated as follows: *bar*, *bar* gene; *Nb-COI1*, the 186 bp fragment of the *Nb-COI1* gene in sense and antisense orientation; INT, the second intron (*IV2*) of the potato *ST-LS1* gene. **(B)** The pLH-*35S-SABP2-INT-SABP2* RNAi construct. The pLH-*35S-SABP2-INT-SABP2* plasmid contains the *bar* and *Nb-SABP2* RNAi expression cassettes. The components of the pLH-*35S-SABP2-INT-SABP2* are designated as follows: bar, *bar* gene; Nb-SABP2, the 230 bp fragment of the *Nb-SABP2* gene in sense and antisense orientation; INT, the second intron (*IV2*) of the potato *ST-LS1* gene. **(C)** The pLH*-TMV-GOI* expression vector. Features are as follows: RdRp, RNA-dependent RNA polymerase; MP, movement protein; genes of interest (GOI): *βNGF* (βNGF) and *GFP* (GFP); CP, coat protein encoding sequence; **(D)** The pLH-*PVX-βNGF* expression vector. Features are as follows: RdRp, RNA-dependent RNA polymerase; 25K, 12K, 8K, triple gene block; βNGF, *βNGF* gene; CP, coat protein encoding sequence. All expression constructs are based on the pLH7000 vector backbone. The regulatory elements used in expression vectors are pAct2, Act2 promoter from *A thaliana*; tNos, nopaline synthase terminator, p35S, CaMV 35S promoter; t35S, CaMV 35S terminator. LB, RB, left and right border of T-DNA, respectively.

**Figure 2:**
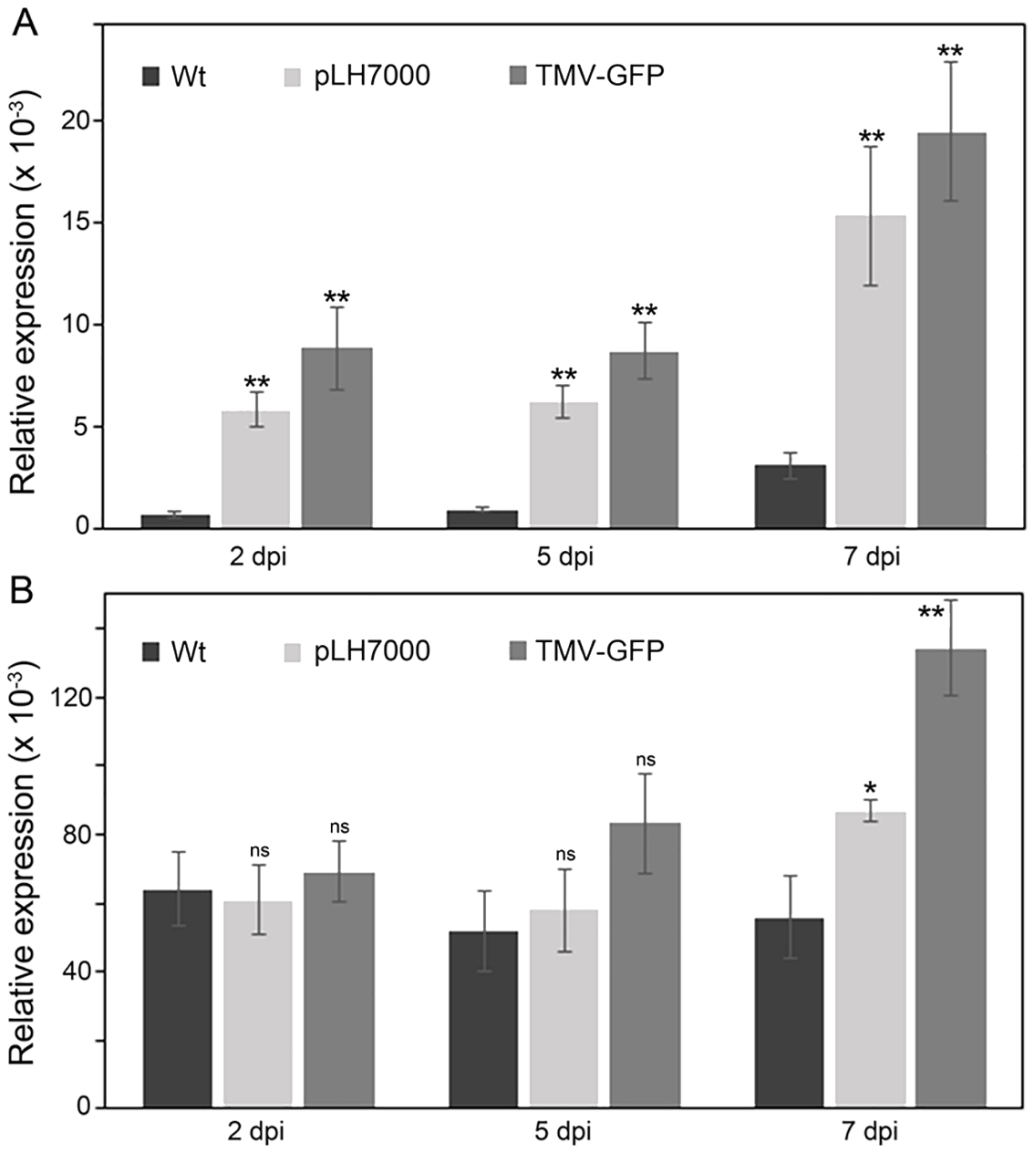
Expression of the *Nb-SABP2* and *Nb-COI1* genes in agroinfiltrated *N. benthamiana* leaves. *N. benthamiana* plants were agroinfiltrated with *A. tumefaciens* cultures carrying the pLH7000 and pLH-*TMV*-*GFP* expression vectors. The total RNA was isolated from leaf samples collected on 2, 5 and 7 dpi and subjected to qPCR analysis. The levels of the *Nb-SABP2* **(A)** and *Nb-COI1* **(B)** mRNA were quantified by gene-specific primers and normalized to *cyp* gene. Values represent the means with SE (n=8). Asterisks indicate significance as determined by the Mann-Whitney test, with *and ** denoting p < 0.05 and p < 0.01, respectively. Not significant values are determined as ns.

To examine the involvement of the *Nb-SABP2* and *Nb-COI1* genes in *N. benthamiana* defense response to Agrobacterium-mediated delivery of virus-based expression vector the expression profile of these genes was investigated in agroinoculated leaf tissue. Following agroinfiltration of *N. benthamiana* plants with *A. tumefaciens* cultures carrying the pLH7000 and pLH-TMV-GFP (**Figure 1**) constructs the *Nb-SABP2* and *Nb-COI1* transcripts were quantified by qPCR at 2, 5 and 7 days after inoculation (dpi) and compared to that of non-treated plants. This analysis showed that the *Nb-SABP2* gene was upregulated in response to *A. tumefaciens* (pLH7000) and TMV (pLH-*TMV-GFP*) at early infection stage, 2 dpi (**Figure 2A**). In the *N. benthamiana* leaves treated only with *A. tumefaciens* the accumulation of the *Nb-SABP2* RNA was increased with a significant fold change value of 5.9 at 2 dpi, 6.3 at 5 dpi and 4.8, at 7 dpi. In presence of the pLH-*TMV-GFP* virus vector the *Nb-SABP2* expression increased at levels 8.9, 8.7 and 6-fold of the means for the non-treated plant at 2, 5 and 7 dpi, respectively. These results suggest that the *Nb-SABP2* gene is involved in *N. benthamiana* defense reaction against both components of transient gene delivery system, Agrobacterium and virus vector, and its upregulation starts at the early infection stage.

The expression profile of the *Nb-COI1* gene differed from that of the *Nb-SABP2* gene (**Figure 2B**). In comparison to non-inoculated control, no significant differences in the *Nb-COI1* transcript accumulation were observed in agroinfiltrated *N. benthamiana* leaves at 2 and 5 dpi. The upregulation of the *Nb-COI1* gene expression in treated leaves started late in the infection, at 7 dpi, achieving a 1.6-fold increase for Agrobacterium (pLH7000) and 2.4-fold increase for TMV (pLH-*TMV-GFP*). Therefore, the *Nb-COI1* gene is also involved in plant defense response against Agrobacterium-mediated delivery of the TMV-based vector.

### Downregulation of the *Nb-SABP2* and *Nb-COI1* genes facilitated TMV and foreign protein transcript accumulation

To determine whether downregulation of the *Nb-SABP2* and *Nb-COI1* endogenous genes can influence the TMV and foreign protein RNA accumulation the pLH-*35S-COI1-INT-COI1* and pLH-*35S-SABP2-INT-SABP2* constructs have been designed. Both construct are based on the pLH7000 vector backbone and utilize the CaMV 35S promoter and terminator to control the dsRNA expression unit. The *Nb-SABP2* dsRNA expression cassette in the pLH-*35S-SABP2-INT-SABP2* vector includes the *Nb-SABP2* DNA fragment of 230 bp in sense and antisense orientation separated by the second intron of the potato *ST-LS1* gene (**Figure 1A**). The pLH-*35S-COI1-INT-COI1* silencing construct contained a 186 bp fragment of the *Nb-COI1* gene in direct and indirect orientation and the same splitting intron sequence as in the case of the pLH-*35S-SABP2-INT-SABP2* plasmid (**Figure 1B**).

**Figure 3:**
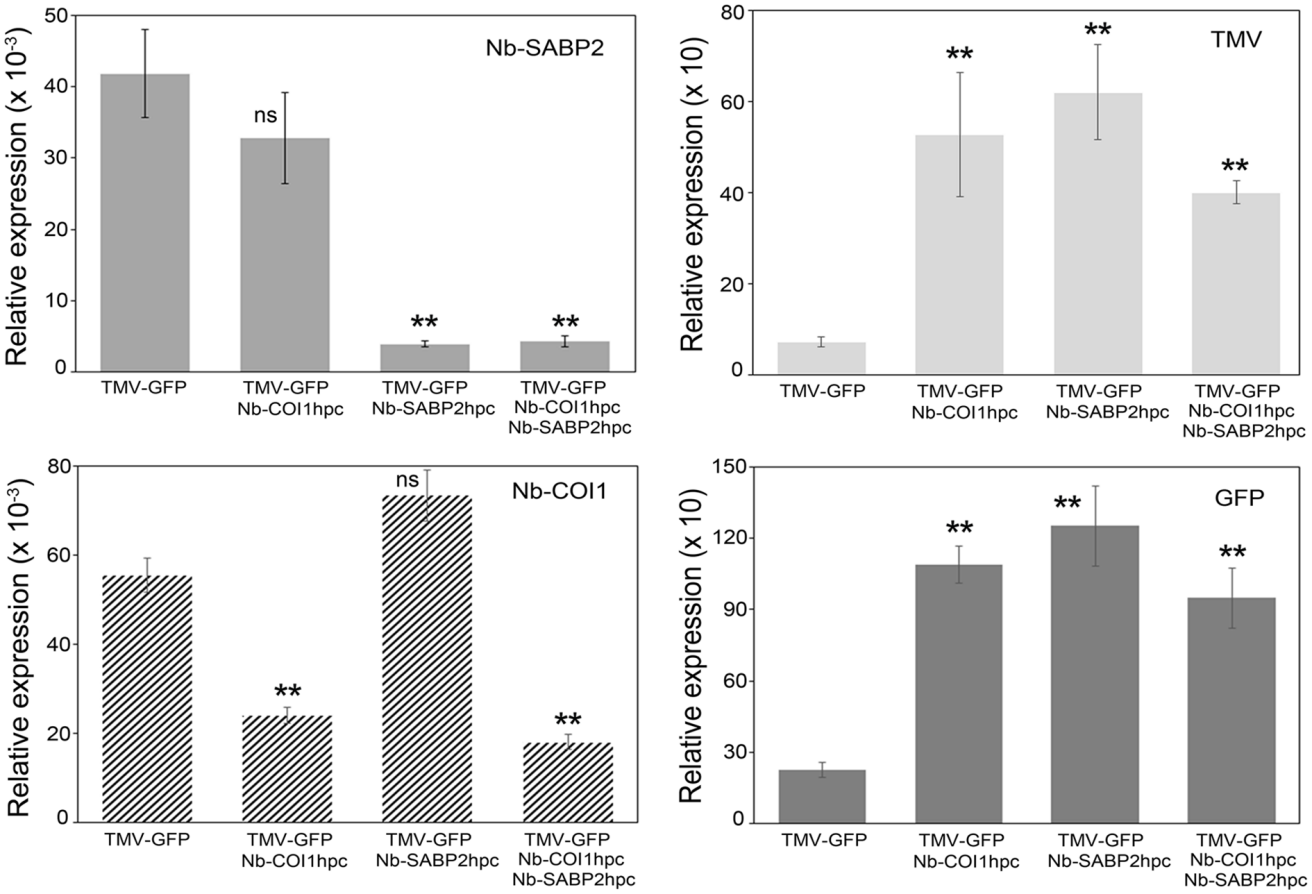
TMV and GFP RNA accumulation in *Nb-COI1* and *Nb-SABP2*-silenced *N. benthamiana* leaves. *N. benthamiana* leaves were agroinfiltrated with four combinations of *A. tumefaciens* cultures, namely, pLH-*TMV-GFP*, pLH-*TMV-GFP*/pLH-*35S-COI1-INT-COI1*, pLH-*TMV-GFP*/pLH-*35S-SABP2-INT-SABP2*, pLH-*TMV-GFP*/pLH-*35S-SABP2-INT-SABP2/*pLH-*35S-COI1-INT-COI1*. Leaf samples for RNA isolation were collected at 6 dpi. RNA levels were quantified by qPCR using primers specific to the *Nb-SABP2*, *Nb-COI1*, TMV and *GFP*. Values represent the means with SE (n=6). Asterisks indicate significance as determined by the Mann-Whitney test, with ** denoting p < 0.01. Not significant values are determined as ns.

The functionality of the pLH-*35S-SABP2-INT-SABP2* and pLH-*35S-COI1-INT-COI1* constructs has been tested for two vectors, pLH-*TMV-GFP* and pLH-*TMV-βNGF* (**Figure 1C**). For each expression vector *N. bethamiana* plants were agroinfiltrated with the *A. tumefaciens* cultures in following combinations: (i) pLH-*TMV-GOI*, (ii) pLH-*TMV-GOI*/pLH-*35S-COI1-INT-COI1*, (iii) pLH-*TMV-GOI/*pLH-*35S-SABP2-INT-SABP2*, (iv) pLH-*TMV-GOI*/pLH-*35S-SABP2-INT-SABP2/*pLH-*35S-COI1-INT-COI1*. The *GOI* designates the *GFP* and *βNGF* encoding sequences. The levels of the *Nb-SABP2*, *Nb-COI1*, TMV, *GFP* and *βNGF* transcripts were determined by qPCR analysis in the *Nb-SABP2* and *Nb-COI1* silenced leaves and compared with those of plants treated only with the pLH-*TMV-GOI* vector. The qPCR results for the pLH-*TMV-GFP* virus vector demonstrated that the *Nb-SABP2* mRNA levels were reduced to 91% and 90% in the pLH-*TMV-GFP*/pLH-*35S-SABP2-INT-SABP2*, and pLH-*TMV-GFP*/pLH-*35S-SABP2-INT-SABP2/*pLH-*35S-COI1-INT-COI1* agroinfiltrated leaves, respectively (**Figure 3**). The presence of the pLH-*35S-COI1-INT-COI1* dsRNA construct resulted in the downregulation of the *Nb-COI1* gene by 57% for the pLH-*TMV-GFP*/pLH-*35S-COI1-INT-COI1* combination and by 68% for the *TMV-GFP*/pLH-*35S-SABP2-INT-SABP2/*pLH*35S-COI1-INT-COI1* mixture of *A. tumefaciens* cultures. The content of TMV RNA elevated by 7, 9 and 6-fold in the *Nb-COI1, Nb-SABP2* and *Nb-COI1*/*Nb-SABP2*-silenced plants, respectively. The accumulation of the *GFP* transcripts correlated with that of the TMV: it was increased by 5, 6 and 4-fold for the pLH-*TMV-GFP*/pLH-*35S-COI1-INT-COI1*, pLH-*TMV-GFP*/pLH-*35S-SABP2-INT-SABP2*, and pLH-*TMV-GFP*/pLH-*35S-SABP2-INT-SABP2/*pLH*35S-COI1-INT-COI1* samples, correspondingly.

**Figure 4:**
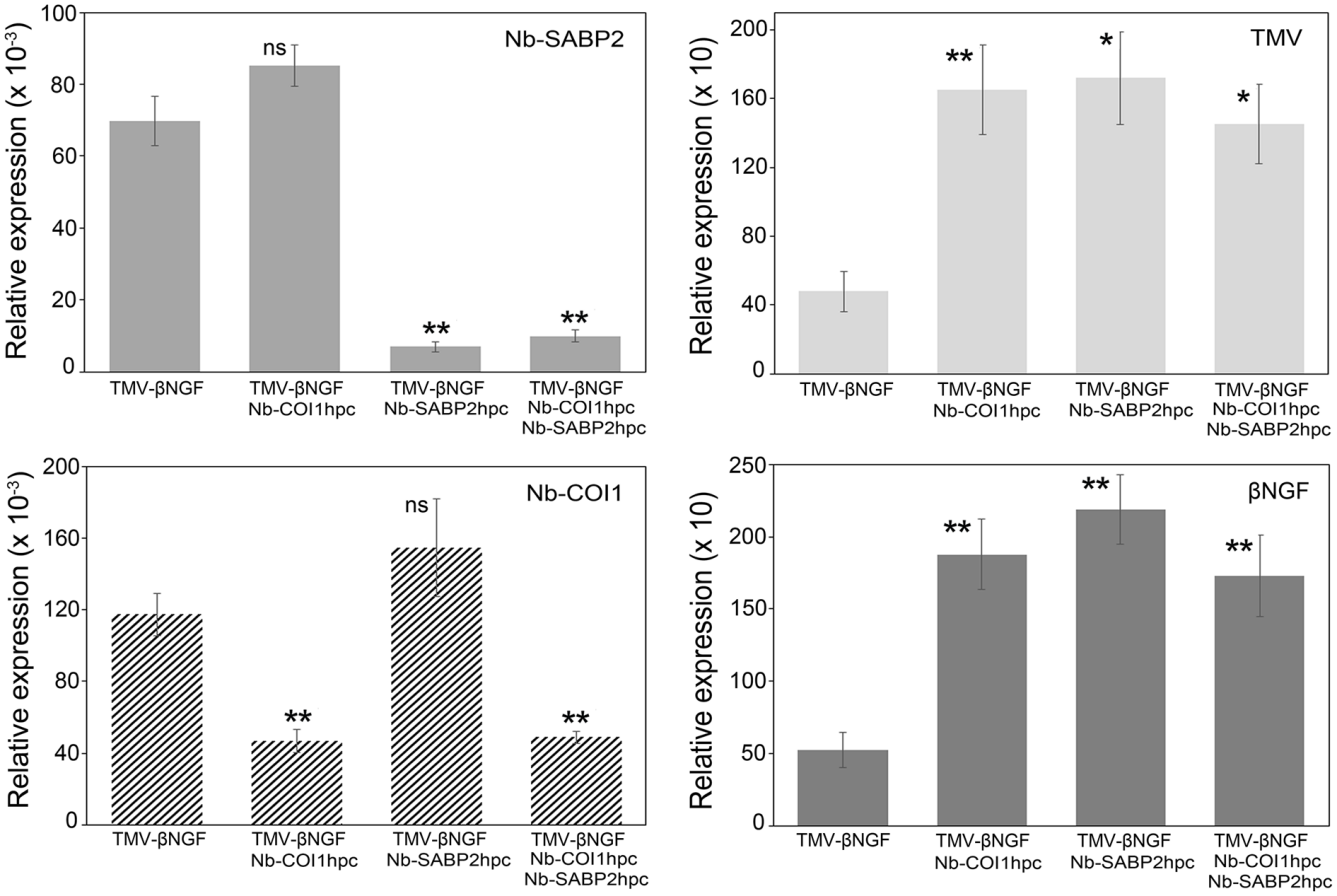
TMV and βNGF RNA accumulation in *Nb-COI1* and *Nb-SABP2*-silenced *N. benthamiana* leaves. *N. benthamiana* leaves were agroinfiltrated with four combinations of *A. tumefaciens* cultures, namely, pLH-*TMV-βNGF*, pLH-*TMV-βNGF*/pLH-*35S-COI1-INT-COI1*, pLH-*TMV-βNGF*/pLH-*35S-SABP2-INT-SABP2*, pLH-*TMV-βNGF*/pLH-*35S-SABP2-INT-SABP2/*pLH-*35S-COI1-INT-COI1*. Leaf samples for RNA isolation were collected at 6 dpi. RNA levels were quantified by qPCR using primers specific to the *Nb-SABP2*, *Nb-COI1*, TMV and *βNGF*. Values represent the means with SE (n=6). Asterisks indicate significance as determined by the Mann-Whitney test, with * and ** denoting p < 0.05 and p < 0.01, respectively. Not significant values are determined as ns.

The qPCR data for the TMV vector carrying the *βNGF* coding sequence are shown in **Figure 4**. Similar to the pLH-*TMV-GFP* virus vector the *Nb-SABP2* expression was reduced by 90% in leaves agroinfiltrated with the pLH-*TMV-βNGF*/pLH-*35S-SABP2-INT-SABP2* culture mixture and by 86% in leaves agroinfiltrated with the pLH-*TMV-βNGF*/pLH-*35S-SABP2-INT-SABP2*/pLH-*35S-COI1-INT-COI1* culture mixture. In leaves agroinfiltrated with the pLH-*TMV-βNGF*/pLH-*35S-COI1-INT-COI1* and pLH-*TMV-βNGF*/pLH-*35S-SABP2-INT-SABP2*/pLH-*35S-COI1-INT-COI1* combinations the *Nb-COI1* mRNA levels were reduced by 60% and 59%, correspondingly. Downregulation of the *Nb-SABP2* and *Nb-COI1* genes resulted in increased accumulation of the TMV RNA at 3 fold in all tested combinations of constructs.

Positive correlation was also found between the accumulation of viral and βNGF RNA. The *βNGF* gene showed significant fold change values of 3.6, 4.2 and 3.3 in the pLH-*TMV-βNGF*/pLH-*35S-COI1-INT-COI1*, pLH-*TMV-βNGF*/pLH-*35S-SABP2-INT-SABP2* and pLH-*TMV-βNGF*/pLH-*35S-SABP2-INT-SABP2*/pLH-*35S-COI1-INT-COI1* samples, respectively. These results suggest that the RNAi constructs for the *Nb-SABP2* and *Nb-COI1* are functional in a transient assay and the suppression of these genes has positive effect on foreign protein transcript accumulation.

### Downregulation of the *Nb-SABP2* and *Nb-COI1* genes enhanced transient recombinant protein accumulation

**Figure 5:**
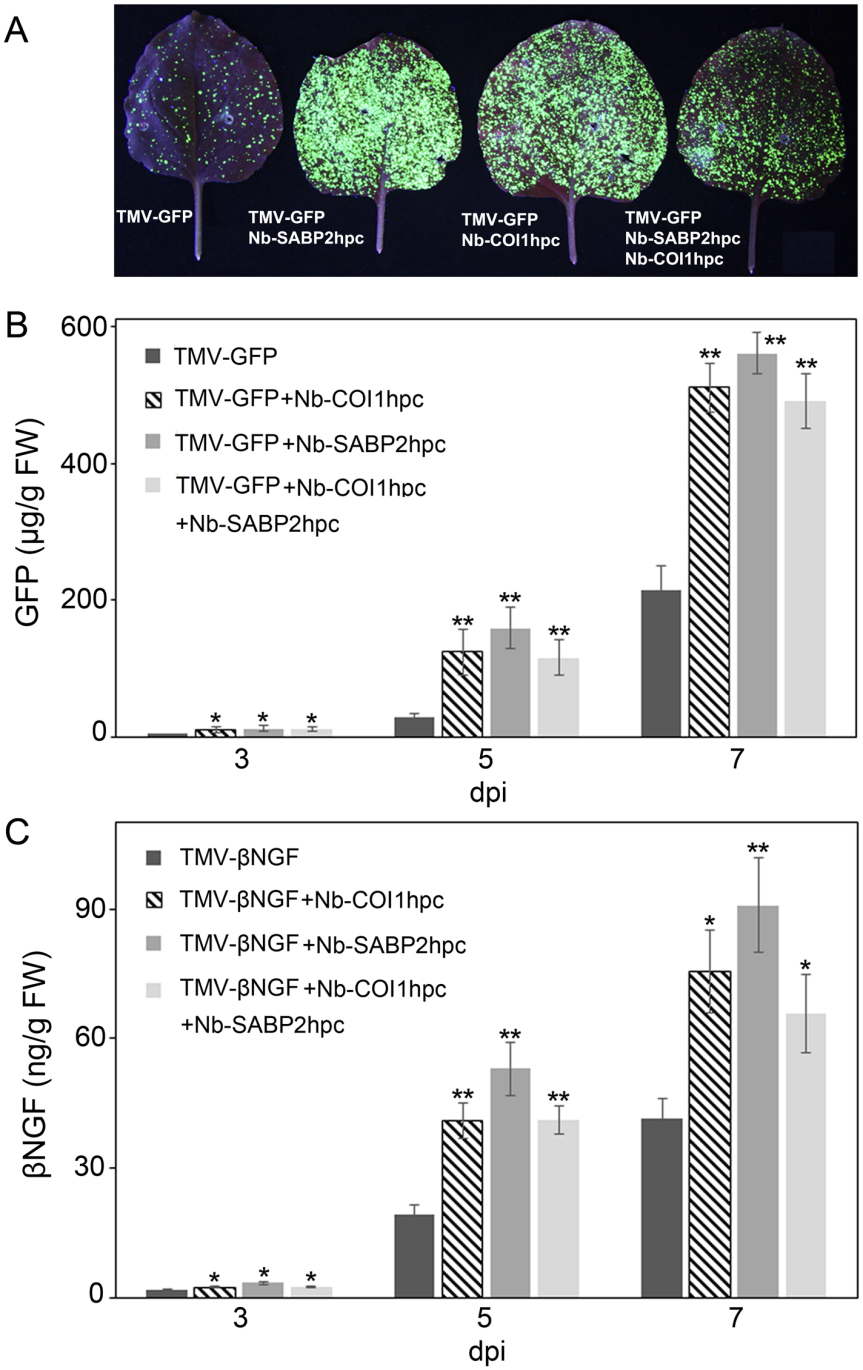
Transient production of GFP and βNGF recombinant proteins in *Nb-COI1* and *Nb-SABP2-*silenced *N. benthamiana* leaves. **(A)** GFP visualization in agroinfiltrated leaves. *N. benthamiana* plants were co-agroinfiltrated with *Nb-COI1* and *Nb-SABP2* RNAi silencing constructs and pLH-*TMV-GFP*. Images were taken at 5 dpi. The representative leaves coinfiltrated with the pLH-*TMV-GFP* (1), pLH-*TMV-GFP*/pLH-*35S-SABP2-INT-SABP2* (2), and pLH-*TMV-GFP*/pLH-*35S-COI1-INT-COI1* (3), pLH-*TMV-GFP*/pLH-*35S-SABP2-INT-SABP2*/pLH-*35S-COI1-INT-COI1* (4) are shown. **(B)** Time course analysis of GFP production. The leaves of *N. benthamiana* plants co-agroinfiltrated with the pLH-*TMV-GFP*, pLH-*TMV-GFP*/pLH-*35S-SABP2-INT-SABP2* and pLH-*TMV-GFP*/pLH-*35S-COI1-INT-COI1*, pLH-*TMV-GFP*/pLH-*35S-SABP2-INT-SABP2*/pLH-*35S-COI1-INT-COI1 A. tumefaciens* cultures were sampled at 3, 5 and 7 dpi and analysed by specific ELISA for GFP accumulation. **(C)** Time course analysis of βNGF production. The leaves of *N. benthamiana* plants co-infiltrated with the pLH-*TMV-βNGF*, pLH-*TMV-βNGF*/pLH-*35S-COI1-INT-COI1*, pLH-*TMV-βNGF*/pLH-*35S-SABP2-INT-SABP2*, pLH-*TMV-βNGF*/pLH-*35S-SABP2-INT-SABP2*/pLH-*35S-COI1-INT-COI1 A. tumefacines* cultures were sampled at 3, 5 and 7 dpi and analysed by specific ELISA for βNGF accumulation. Values represent the means with SE (n=8). Asterisks indicate significance as determined by the Mann-Whitney test, with * and ** denoting p < 0.05 and p < 0.01, respectively.

Having identified the beneficial effect of the *Nb-SABP2* and *Nb-COI1* gene suppression on transient gene delivery system at RNA level (**Figures 3**, **4**), we assessed the recombinant protein accumulation. Investigation of *N. benthamiana* leaves agroinfiltrated with the pLH-*TMV-GFP* under UV light revealed stronger GFP fluorescence in the presence of the *Nb-SABP2* and *Nb-COI1* RNAi silencing constructs (**Figure 5A**). Following a visual observation, the expression of GFP recombinant protein was analyzed over time in *N. benthamiana* leaves by specific ELISA. This analysis showed that the GFP accumulation increased steadily from day 3 to 7 and reached a peak on day 7 (**Figure 5B**). Compared to samples agroinfiltrated with the pLH*-TMV-GFP* alone, higher recombinant protein amounts were detected in leaf tissue co-infiltrated with the *A. tumefaciens* cultures containing the pLH-*TMV-GFP* and RNAi expression vectors. Production of GFP increased by 2.4-fold for the pLH-*TMV-GFP*/pLH-*35S-COI1-INT-COI1* combination of expression constructs, by 2.6-fold for the pLH-*TMV-GFP*/pLH-*35S-SABP2-INT-SABP2* combination of expression constructs and by 2.2-fold for the pLH-*TMV-GFP*/pLH-*35S-SABP2-INT-SABP2*/pLH-*35S-COI1-INT-COI1* combination of expression constructs. The highest GFP accumulation level 560 ± 30 µg/g fresh weight was detected at 7 dpi in the *N. benthamiana* leaves co-infiltrated with the pLH-*35S-SABP2-INT-SABP2* RNAi construct.

The expression profile of βNGF protein was similar to that of the GFP protein with the accumulation peak at 7 dpi (**Figure 5C**). The ELISA data revealed that co-expression of the pLH-*TMV-βNGF*/pLH-*35S-COI1-INT-COI1*, pLH-*TMV-βNGF*/pLH-*35S-SABP2-INT-SABP2* and pLH-*TMV-βNGF*/pLH-*35S-SABP2-INT-SABP2*/pLH-*35S-COI1-INT-COI1* constructs increased βNGF accumulation over the pLH-*TMV-βNGF* control in 1.8, 2.2 and 1.6-folds, respectively. The highest βNGF production 91 ± 10 ng/g fresh weight was observed at 7 dpi when the pLH-*TMV-βNGF* and pLH-*35S-SABP2-INT-SABP2* were combined.

**Figure 6:**
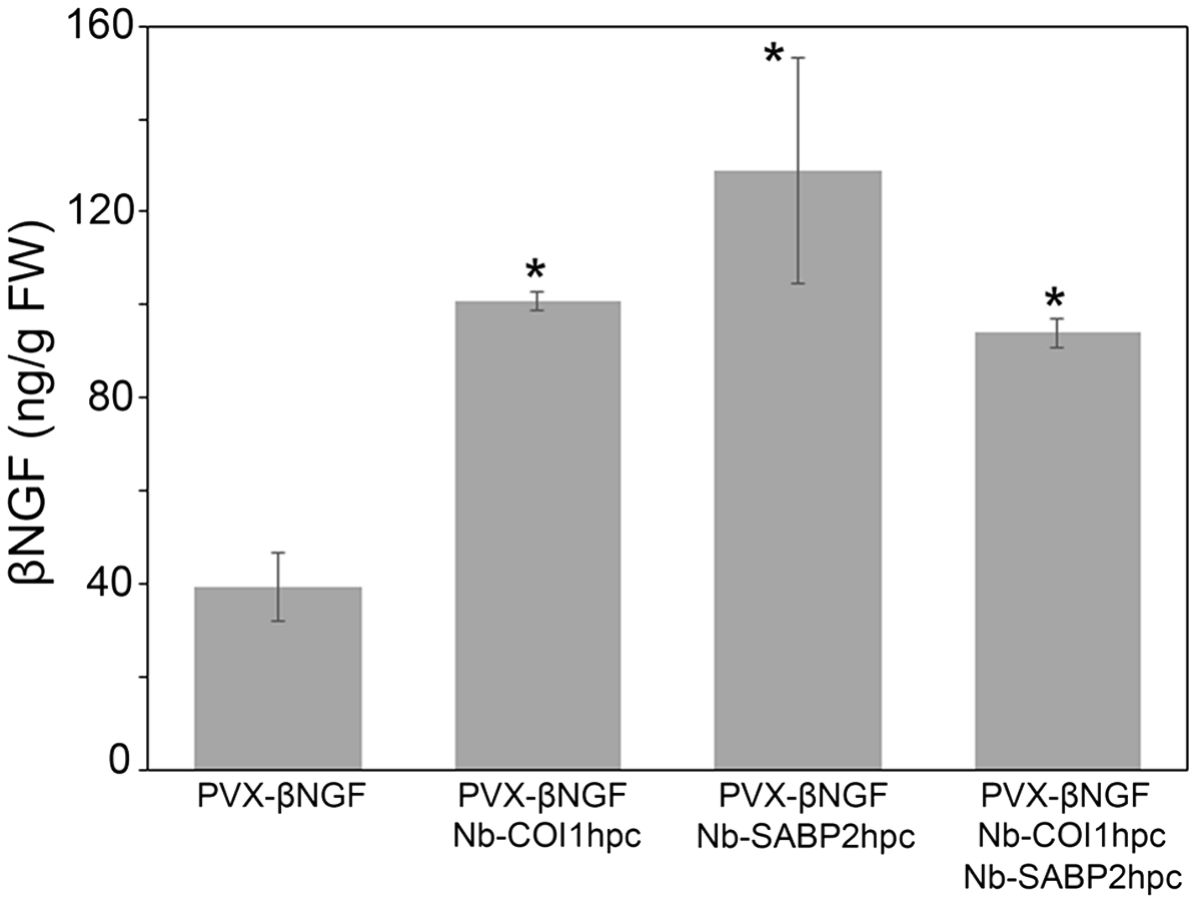
Transient production of the βNGF recombinant protein delivered by the PVX-based vector in the *Nb-COI1* and *Nb-SABP2*-silenced *N. benthamiana* leaves. The leaves of *N. benthamiana* plants co-infiltrated with the pLH-*PVX-βNGF*, pLH-*PVX-βNGF*/pLH-*35S-COI1-INT-COI1*, pLH-*PVX-βNGF*/pLH-*35S-SABP2-INT-SABP2*, pLH-*PVX-βNGF*/pLH-*35S-SABP2-INT-SABP2*/pLH-*35S-COI1-INT-COI1 A. tumefacines* cultures were sampled at 5 dpi and analyzed by specific ELISA for βNGF accumulation. Values represent the means with SE (n=6). Asterisks indicate significance as determined by the Mann-Whitney test, with * denoting p < 0.05.

To investigate whether RNAi-mediated suppression of the *Nb-SABP2* and *Nb-COI1* plant defense related genes can facilitate the accumulation of βNGF delivered by the PVX-based expression vector *N. benthamiana* plants were agroinfiltrated with the *A. tumefaciens* cultures carrying following constructs: the pLH-*PVX-βNGF*/pLH-*35S-COI1-INT-COI1*, pLH-*PVX-βNGF*/pLH-*35S-SABP2-INT-SABP2* and pLH-*PVX-βNGF*/pLH-*35S-SABP2-INT-SABP2*/pLH-*35S-COI1-INT-COI1*. Five days after inoculation the agroinfiltrated leaves were examined using ELISA assay to determine the βNGF production. The results of this analysis are shown in **Figure 6**. In line with the results obtained for the pLH-*TMV-βNGF* expression vector knockdown of the *Nb-SABP2* and *Nb-COI1* genes had positive impact on the βNGF accumulation. Co-infiltration of the pLH-*35S-COI1-INT-COI1* caused 2.6-fold enhancement of the βNGF amount. Combination of the pLH-*PVX-βNGF* and pLH-*35S-SABP2-INT-SABP2* led to 3.3-fold increase in βNGF content. Finally, simultaneous suppression of *Nb-SABP2* and *Nb-COI1* gene expression by the corresponding RNAi constructs resulted in 2.4 fold increase in βNGF accumulation. The best performing combination (pLH-*PVX-βNGF*/pLH-*35S-SABP2-INT-SABP2*) yielded 129 ± 25 ng/g fresh weight of βNGF.

The size and relative abundance of GFP and βNGF were estimated using Western blot analysis in total protein extracts from agroinfiltrated plant tissue. In the *Nb-SABP2* and *Nb-COI1* downregulated plants as well as in the control plants agoinfiltrated only with the pLH-*PVX-βNGF* and pLH-*TMV-GFP* the produced proteins had the expected size of 27 kDa for GFP and 35-37 kDa for βNGF (**Supplementary Figure S1**).

## Discussion

The success of transient expression depends on both host plant and expression vector delivery component. Recent overview of the molecular changes in agroinfiltrated *N. benthamiana* leaves demonstrated that hybrid expression systems consisting of *A. tumefaciens* and binary vector trigger the plant defense response and this can limit the recombinant protein yield (Hamel et al., 2023). In this study we suppressed *N. benthamiana* immunity through knockdown of endogenous genes involved in plant hormone metabolism. The expression pattern of the *Nb-SABP2* and *Nb-COI1* genes assessed by qPCR analysis revealed that both genes were upregulated upon agroinfiltration conferring their involvement in plant immune reaction. The association of the SABP2 with SA-dependent plant defense response to biotic stress has been shown by several research groups. In *Nicotiana tabacum* the *Nt-SABP2* gene expression was induced after TMV inoculation (Kumar and Klessig, 2003). Similarly, the functional homologs of the *Nt-SABP2* gene from *A. thaliana*, *At-MES1*, *At-MES7* and *At-MES9* were transcriptionally upregulated during infection with *Pseudomonas syringae* (Vlot et al., 2008). Our data are also consistent with the experiments demonstrating the positive role of a key regulator of JA signaling COI1 in plant defense. For example, in *Sacharum sp* the expression levels of the *Ss-COI1–4b* and *Ss-COI1–3b* genes were increased in smut-resistant cultivar YC05–179 and downregulated in smut-susceptible cultivar ROC22 (Sun et al., 2022). Similarly, the higher *Ta-COI1* transcript amounts were observed during early response to *Blumeria graminis* in resistant *Triticum aestivum* variety in comparison to susceptible one (Liu et al., 2018).

To examine whether suppression of plant immune system can facilitate the TMV multiplication we targeted the *Nb-SABP2* and *Nb-COI1* genes with corresponding dsRNAs. Downregulation of these genes increased susceptibility to TMV virus carrying *GFP* and *βNGF* sequences, which was manifested by the enhanced viral RNA accumulation in agroinfiltrated leaves. Our data are in agreement with the previous reports demonstrating that SA functions as a positive regulator of TMV resistance. For instance, Kumar and Klessig (2003) showed that the *Nt-SABP2*-silenced *N. tabacum* plants accumulated higher levels of TMV coat protein in comparison to control plants. In *N. benthamiana* knockdown of SA-metabolism-related genes including *Nb-SABP2*, *Nb-ICS1* (isochorismate synthase), *Nb-NPR1* (non-expresser of PR gene 1), *Nb-SAMT* (SA methyl transferase) strongly increased susceptibility to TMV (Zhu et al., 2014). The role of JA in TMV resistance has not been fully clarified. Our data are in accordance with the report showing the positive role of the JA biosynthetic and signaling genes in antiviral response: downregulation of the *Nb-COI1*, *Nb-OPR3* (12-oxo-phytodienoic acid reductase), *NbJMT* (jasmonic acid carboxyl methyltransferase) genes boosted accumulation of TMV (Zhu et al., 2014). In contrast, Oka and co-workers demonstrated that silencing of the JA receptor COI1 resulted in resistance against TMV in tobacco containing N gene (Oka et al., 2013). These authors suggested that the JA signaling is indirectly responsible for TMV susceptibility through modification of SA-dependent and SA-independent resistance pathways.

The usefulness of strategy based on plant defense response modification for biotechnological purposes was demonstrated for GFP and βNGF recombinant proteins: the enhanced accumulation of TMV correlated with the increased amounts of GFP and βNGF transcripts. Furthermore, ELISA investigation confirmed this observation at protein level. For TMV-based expression vector, the downregulation of the *Nb-SABP2*, *Nb-COI1* and *Nb-SABP2*/*Nb-COI1* genes enhanced GFP accumulation by 2.4, 2.6 and 2.2 fold, respectively. Co-expression of the pLH-*TMV-βNGF* and RNAi constructs increased βNGF yield by 1.8-fold for the pLH-*TMV-βNGF*/pLH-*35S-COI1-INT-COI1* combination, 2.2-fold for the pLH-TMV*-βNGF*/pLH-*35S-SABP2-INT-SABP2* combination and 1.6-fold for the pLH-*TMV-βNGF*/pLH-*35S-SABP2-INT-SABP2*/pLH-*35S-COI1-INT-COI1* combination. These results are in content with data that have been reported for a number of strategies for plant host optimization. In particular, transient co-delivery of virus-derived cell cycle regulator genes elevated GUS accumulation about 2-3 fold (Norkunas et al., 2018). Similar increase in production of GFP and scFv-TM43-E10 antibody fragment was observed in *N. benthamiana* leaves, which were co-infiltrated with the At-CycD2 and At-CDC27a plant cell regulators and TMV-based vector containing the *GFP* and *scFvTM43-E10* sequences (Kopertekh and Schiemann, 2019). Modification of gene silencing machinery through simultaneous knockout of *Nb-DCL2* and *Nb-DCL4* genes using CRISPR/Cas9 technology enhanced the production of human fibroblast growth factor by 9 fold (Matsuo, 2022). In the same manner, the CRISPR/Cas9-mediated editing of the *Nb-RDR6* gene elevated GFP accumulation in *N. benthamiana* by 2.5-fold (Matsuo and Atsumi, 2019).

A combined downregulation of multiple genes may have an additive effect to maximize the recombinant protein yield in transient expression. The simultaneous silencing of the *Nb-SABP2* and *Nb-COI1* genes did not result in synergistic effect compared to the suppression of the *Nb-SABP2* and *Nb-COI1* alone: we observed lower RNA and protein accumulation levels for TMV, GFP and βNGF in double-silenced plants. Data reported in several papers indicated the importance of the crosstalk between JA and SA to determine the degree of TMV resistance (Oka et al., 2013; Zhu et al., 2014). It can be suggested that in the *Nb-SABP2/Nb-COI1-*suppressed leaves the balance between endogenous JA and SA did not provide the superior cellular conditions for TMV infection in comparison to the individual silencing of the *Nb-SABP2* and *Nb-COI1* genes. Therefore, separated knockdown of the *Nb-SABP2* and *Nb-COI1* genes is more effective for enhanced recombinant protein production.

In summary, we demonstrated in this study that the components of Agrobacterium-mediated vector delivery system upregulate the *Nb-SABP2* and *Nb-COI1* genes, which take part in SA and JA-mediated plant defense response. The suppression of plant immunity through silencing of these genes improved recombinant protein yield as was exemplified by the full TMV-based expression vector. We suppose that this strategy can be useful for several commercial transient expression systems including GENEWARE^®^ (Shivprasad et al., 1999), TRBO (Lindbo, 2007), magnICON^®^ (Gleba et al., 2007), TMV launch vector (Musiychuk et al., 2007) utilizing TMV as the vector backbone. This approach might be extended to other virus-based expression vectors given that other plant-virus-based expression systems are limited by the SA- and JA-mediated virus resistance. Indeed, we demonstrated the increased βNGF production in the presence of the *Nb-SABP2* and *Nb-COI1* RNAi silencing constructs when the *βNGF* encoding sequence was delivered by the PVX-based virus vector.

It is important to note that this strategy is might be incompatible with the application of expression vectors containing strong suppressors of gene silencing such as P19 protein of Tomato bushy stunt virus (TBSV) (Scholthof, 2006). However, the commercial plant virus-based expression vectors listed above do not utilize strong silencing suppressors.

## Data Availability

The raw data supporting the conclusions of this article will be made available by the author, without undue reservation.
