# Supplementary material for: Improving transient expression in N. benthamiana by suppression of the Nb-SABP2 and Nb-COI1 plant defence response related genes
Source: Front Plant Sci. 2024 Sep 9;15:1453930. doi: 10.3389/fpls.2024.1453930 (PMC11416979; doi:10.3389/fpls.2024.1453930)
Supplement: Supplementary file 1 [file DataSheet1.pdf]

SUPPLEMENTARY MATERIAL

FIGURES

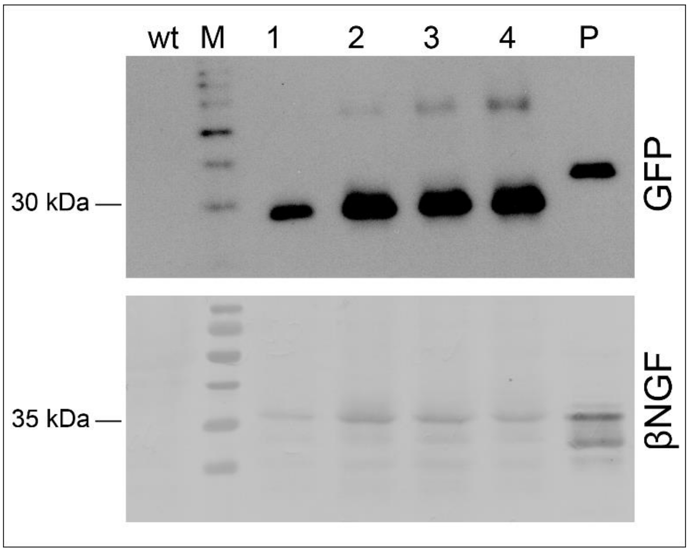

Figure S1. Transient expression of the GFP and β-NGF recombinant proteins in *N. benthamiana* plants. *N. bentamiana* leaves were agroinfiltrated with the pLH-*TMV-GFP* and *pLH-PVX-βNGF* expression vectors in combination with the *Nb-COI1* and *Nb-SABP2* RNAi silencing constructs. Leaf protein extracts (7 and 20 µg of TSP for pLH-*TMV-GFP* and *pLH-PVX-βNGF*, respectively) were separated on reduced 12% SDS-PAGE gel and probed with the antibodies specific to GFP and β-NGF. Lanes are as follows: 1, leaves agroinfiltrated with the pLH-*TMV-GFP* and *pLH-PVX-βNGF*; 2, leaves agroinfiltrated with the pLH-*TMV-GFP* and *pLH-PVX-βNGF* in combination with the pLH-*35S-COI1-INT-COI1*; 3, leaves agroinfiltrated with the pLH-*TMV-GFP* and *pLH-PVX-βNGF* in combination with the pLH-*35S-SABP2-INT-SABP2*; 4, leaves agroinfiltrated with the pLH-*TMV-GFP* and *pLH-PVX-βNGF* in combination with the pLH-*35S-SABP2-INT-SABP2*/pLH-*35S-COI1-INT-COI1*. Wt, non-inoculated *N. benthamiana plant*. M, protein molecular marker. P, GFP and β-NGF recombinant proteins.

METHODS

Plant samples (100 mg) were collected from the middle agroinfiltrated leaves at 5 dpi. Each sample was pooled from three leaves of one plant. Leaf tissue was ground in 400 µl of 250 mM Tris-HCI (pH 7-8). After adding 400 µl of loading buffer (125 mM Tris-HCI, pH 6.8, 10% sodium dodecyl sulfate, 25% glycerol and 12,5% ß-mercaptoethanol), the samples were boiled for 10 min and then centrifuged to remove all insoluble material. Proteins were separated on 12% SDS polyacrylamide gel and then transferred to the immobilon-P-membrane (Amersham). The detection of GFP by the GE Healthcare Amersham™ ECL Prime Western blotting detection reagent has been described previously (Kopertekh et al. 2004). The β-NGF bands were visualized using specific polyclonal antibodies. Binding of the β-NGF primary antibodies was detected using the anti-rabbit alkaline phosphatase antibodies. Finally, the BCIP/NBT substrate (Sigma) system was used in the reactions according to the manufacturer's protocols.

REFERENCES

Kopertekh, L., Juttner, G., Schiemann, J. (2004). Site-specific recombination induced in transgenic plants by PVX virus vector expressing bacteriophage P1 recombinase. *Plant Sci* 166, 485-492. doi: 10.1016/j.plantsci.2003.10.018

TABLES

Table S1. Primers used in this study. Recognition sites for restriction enzymes used for cloning are underlined.

Primer	Sequence (5'-3')	Application
NcoI-COI1-forw	GCCCCATGGAGATCTGCCACTTGA	Cloning
COI1-BamHI-rev	GACGGATCCTCTAGAAGGCCTTCA	Cloning
SpeI-COI1-forw	GCCACTAGTAGATCTGCCACTTGA	Cloning
COI1-XhoI-rev	GACCTCGAGTCTAGAAGGCCTTCA	Cloning
NcoI-SABP2-forw	GGTCCATGGGTTCCCCGTAAGAGC	Cloning
SABP2-HindIII-rev	CCGAAGCTTTTCTGGTATGGCTTT	Cloning
XbaI-SABP2-forw	GGATCTAGAGTTCCCCTGAAGAGC	Cloning
SABP2-SalI-rev	CCGGTCGACTTCTGGTATGGCTTT	Cloning
BglII-ST-LS1-INT-forw	GGCGAGATCTGTAAGTTTCTGCTCT	Cloning
HindlII-ST-LS1-INT-forw	GGCGAAGCTTGTAAGTTTCTGCTCT	Cloning
ST-LS1-INT-SalI-rev	GGATGTCGACCTGCACATCAACAAA	Cloning
Cyp-forw	CCGACGAGAACTTCAAGAGG	qPCR
Cyp-rev	CCACAGTCAGCAACCACAAC	qPCR
Nb-SABP2-forw	GCTAGAAGGTGCAGGCCATA	qPCR
Nb-SABP2-rev	CAAGACTATGCCCCACCAAT	qPCR
Nb-COI1-forw	TGCATGAACTAGCGGTGAAC	qPCR
Nb-COI1-rev	AATGAGCCACCACCAAACTC	qPCR
TMV-CP-forw	AGATCCTGTGCAGCTGATCA	qPCR
TMV-CP-rev	TAGTATTCGGTGCGGGTTGA	qPCR
gfp-forw	GAAGCAGCACGACTTCTTCA	qPCR
gfp-rev	CGGCCATGATATAGACGTTG	qPCR
βNGF-forw	AGGTGATGGTGTTGGGAGAG	qPCR
βNGF-rev	GACGCACACACAGGCAGTAT	qPCR
